# The dinosaurian femoral head experienced a morphogenetic shift from torsion to growth along the avian stem

**DOI:** 10.1098/rspb.2022.0740

**Published:** 2022-10-12

**Authors:** Shiro Egawa, Christopher T. Griffin, Peter J. Bishop, Romain Pintore, Henry P. Tsai, João F. Botelho, Daniel Smith-Paredes, Shigeru Kuratani, Mark A. Norell, Sterling J. Nesbitt, John R. Hutchinson, Bhart-Anjan S. Bhullar

**Affiliations:** ^1^ Department of Earth & Planetary Sciences and Peabody Museum of Natural History, Yale University, New Haven, CT 06520, USA; ^2^ Laboratory for Evolutionary Morphology, RIKEN Center for Biosystems Dynamics Research (BDR), Kobe, Japan; ^3^ Department of Neurophysiology, National Institute of Neuroscience, National Center of Neurology and Psychiatry, Tokyo, Japan; ^4^ Structure and Motion Laboratory, Department of Comparative Biomedical Sciences, The Royal Veterinary College, Hawkshead Lane, North Mymms AL9 7TA, UK; ^5^ Museum of Comparative Zoology, Department of Organismic and Evolutionary Biology, Harvard University, Cambridge, MA, USA; ^6^ Geosciences Program, Queensland Museum, Brisbane, Australia; ^7^ Mécanismes adaptatifs et évolution (MECADEV)/UMR 7179, CNRS/Muséum National d'Histoire Naturelle, Paris, France; ^8^ Department of Biomedical Sciences, Missouri State University, Springfield, MO 65897, USA; ^9^ Department of Biology, Southern Connecticut State University, New Haven, CT 06515, USA; ^10^ Escuela de Medicina Veterinaria, Facultad de Agronomía e Ingeniería Forestal, Facultad de Ciencias Biológicas y Facultad de Medicina, Pontificia Universidad Católica de Chile, Santiago, Chile; ^11^ Division of Vertebrate Paleontology, American Museum of Natural History, New York, NY, USA; ^12^ Department of Geosciences, Virginia Tech, Blacksburg, VA 24061, USA

**Keywords:** developmental evolution, dinosaur, locomotor apparatus, morphogenesis, homology, developmental system drift

## Abstract

Significant evolutionary shifts in locomotor behaviour often involve comparatively subtle anatomical transitions. For dinosaurian and avian evolution, medial overhang of the proximal femur has been central to discussions. However, there is an apparent conflict with regard to the evolutionary origin of the dinosaurian femoral head, with neontological and palaeontological data suggesting seemingly incongruent hypotheses. To reconcile this, we reconstructed the evolutionary history of morphogenesis of the proximal end of the femur from early archosaurs to crown birds. Embryological comparison of living archosaurs (crocodylians and birds) suggests the acquisition of the greater overhang of the femoral head in dinosaurs results from additional growth of the proximal end in the medial-ward direction. On the other hand, the fossil record suggests that this overhang was acquired by torsion of the proximal end, which projected in a more rostral direction ancestrally. We reconcile this apparent conflict by inferring that the medial overhang of the dinosaur femoral head was initially acquired by torsion, which was then superseded by mediad growth. Details of anatomical shifts in fossil forms support this hypothesis, and their biomechanical implications are congruent with the general consensus regarding broader morpho-functional evolution on the avian stem.

## 1. Introduction

**Figure 1.**
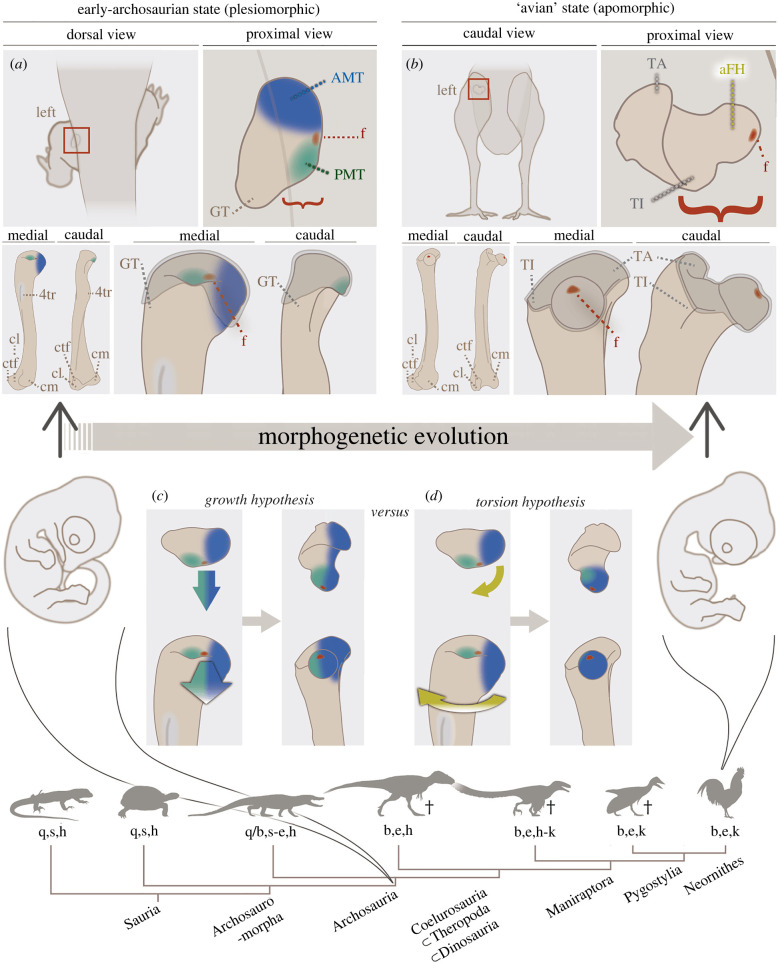
Archosaur femoral head: comparison of the medial-ward overhangs and ‘conflicting’ hypotheses to derive the apomorphic state. The left femora highlighted; (*a*) the plesiomorphic state seen in early diverging archosaurs such as extant crocodylians and (*b*) the apomorphic state seen in extant birds, traced from specimens of figure 4 (*a*) and (*h*), respectively. Red brackets indicate the degree of the femoral head overhang towards the body (i.e. medial-ward). The left bottom panels are magnified in the right bottom ones with the proximal ends highlighted. aFH: ‘avian’ femoral head offset, AMT (blue): antero-medial tuber, cl: condylus lateralis, cm: condylus medialis, f (red): fovea, GT: ‘greater trochanter’, PMT (green): postero-medial tuber, TA: trochanter major, TI: trochanter minor, 4tr: 4th trochanter. Grey shading represents areas within synovial capsules [20,21]. (*c,d*) Two hypotheses have been proposed for the evolutionary transition from plesiomorphic to apomorphic state (i.e. acquisition of the greater medial overhang of the proximal end). The growth hypothesis (*c*) proposes the mediad growth of the medial region of the proximal end. The torsion hypothesis (*d*) proposes the twist of the proximal end against the more distal region. The cladogram is simplified from electronic supplementary material, figure S1 with locomotory modes; quadru-/bi-pedal (q/b), sprawling∼intermediate∼erect posture (s/i/e), hip-/knee-driven (h/k). ‘†’ indicates extinct taxa. Silhouettes are from Phylopic (copyrights, from left to right; Zdenek CN, Farke AA, Hartman S, Chávez C, Vladimir W, Hartman S and Traver S). (Online version in colour.)

Dinosauria is one of the most anatomically distinctive clades in Archosauria, which is today represented by extant dinosaurs (birds) and their closest relatives (crocodylians). Dinosaurian evolution and diversification have long been discussed with special attention given to locomotion (e.g. erect posture, parasagittal gait and gigantism/miniaturization), in which the hip musculo-skeletal system played a central role [1–19]. The tetrapod hip joint consists of an acetabular cavity/foramen in the pelvis and the proximal end of the femur articulating with each other. Ancestrally in Archosauria, the proximal end of the femur was only slightly offset from the femoral shaft; this state is retained in crocodylians (see below for discussion on the evolutionary polarity of the character state) (figure 1*a*). In later dinosaurs, including birds, the prominent femoral head overhangs the shaft mediad (figure 1*b*), which has long been recognized as an unusual characteristic for reptiles (e.g. [22]) and has been considered to be the key feature for dinosaurian locomotor specializations (so-called ‘buttress erect posture’ [2,3,6]).

There are two conflicting hypotheses for the derivation of the prominent overhang of the dinosaurian femoral head. One hypothesis is that the medial region of the femur simply extended mediad towards the acetabulum without torsion (‘growth hypothesis’, figure 1*c*). The exact region that is hypothesized to have undergone inward extension varies; the region around the insertion site of ligamentum capitis femoris (the fovea) [6,9]; or a region called the postero-medial tuber (PMT) [23] (figure 1*a,b*). Another hypothesis is that the antero-medially projected proximal end of the femur became more medially oriented through femoral body torsion (‘torsion hypothesis’, figure 1*d*) [13,23–25]). This conflict in interpretation stems mainly from an exclusive emphasis on either neontological or palaeontological evidence of adult morphology.

Similar disputes between neontology and palaeontology have been resolved successfully by the detailed integration of data from both sources, considering the evolution of morphogenesis (i.e. the developmental process of form), through which morphological evolution necessarily occurs (e.g. hand digit homology of birds and other theropods; [26–29]). Thus, we reconstructed the evolutionary history of femoral head morphogenesis on the line to crown group birds (Neornithes) with the goal of reconciling these apparently incommensurable hypotheses. There are two key data sources to infer morphogenesis: embryos of extant clades closely related to the targeted group as well as outgroups, and fossils informative for its morphogenesis. Hence, to take a total-evidence approach, we draw from both sources and construct a reconciling hypothesis.

## 2. Material and methods

Embryos were harvested and staged according to [30] (chicken *Gallus gallus* and quail *Coturnix japonica*), [31] (alligator *Alligator mississippiensis*), [32] (turtle *Pelodiscus sinensis*) and [33] (gecko *Paroedura picta*). Embryonic skeletons were three-dimensionally visualized by modified CLARITY protocol [34] except for electronic supplementary material, figure S5, which was visualized by section-based reconstruction with Avizo. Dye labellings were performed with CM-DiI (whole embryo, quail) or PKH-26 (organ culture on chorio-allantoic membrane, chicken).

Femoral head angle of osteological specimens was defined as the one between the ‘distal end axis’ (see electronic supplementary material) and the long axis of the proximal end. The angles were measured in orthographic distal views of three-dimensional data. The angle values, if available, were averaged between left and right femora from each individual, then within each species, and finally within each genus. Measurements are summarized in electronic supplementary material, table S1.

See the electronic supplementary material for the full details.

## 3. Result and discussion

### (a) Embryology (neontology) supports the growth hypothesis

**Figure 2.**
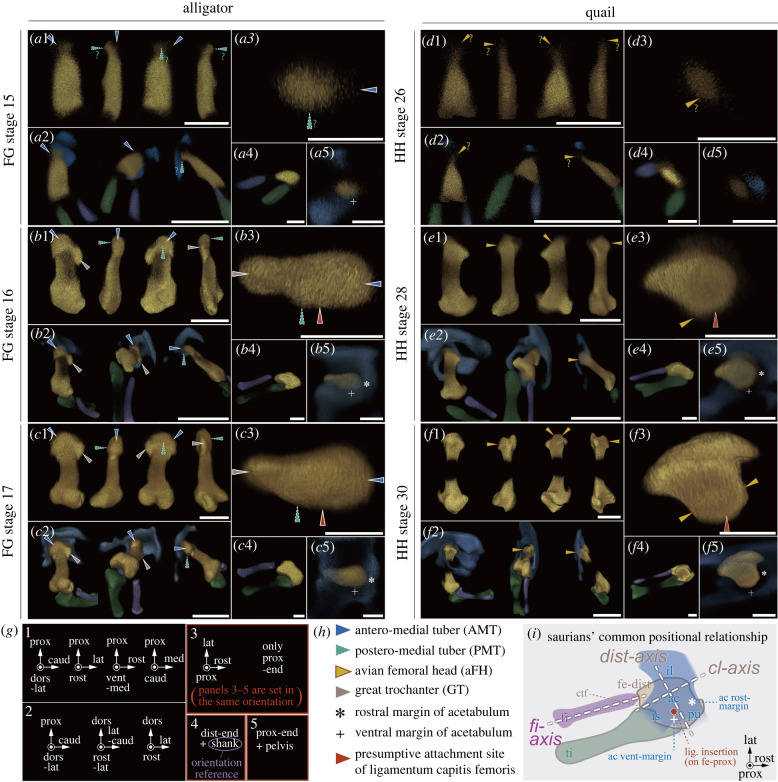
Neontology: comparison of femoral head morphogenesis in extant archosaurs. Cartilaginous skeletons of embryonic hindlimbs are visualized in alligator (*Alligator mississippiensis, a–c*) and quail (*Coturnix japonica, d–f*), with the youngest stage on the top. All the skeletons are left ones or mirrored right ones. Blue, pelvis; Golden, femur; Green, tibia; Purple, fibula. All of these are in pseudo-colours. Skeletons are aligned according to orientations in (*g*). 3–5 of each panel (e.g. *a*3, *a*4, *a*5 and so on) shows proximal views with the strictly same orientation within each individual. 3 s show only the proximal end of the femora (approximately distal two-thirds are not displayed). 4 s show the distal end of the femora (approximately proximal two-thirds are not displayed) and the zeugopods (tibiae and fibulae) to demonstrate that fibular orientations are aligned among all individuals with different developmental stages. 5 s show the proximal end of the femora and translucently visualized pelves. figure 1*a,b* for the details of anatomical landmarks captioned in (*h*). (*i*) schematizes the common positional relationship for saurian embryonic skeleton (including gecko and turtle, electronic supplementary material, figure S2) with conservative orientations among the fibula (fi-axis), the lateral condyle of the distal end of the femur (cl-axis; cf. ‘dist-axis’ is defined as perpendicular to it), and the acetabulum, which can be recognized with the positions of the ventral (+) and rostral (*) margins. Note that the presumptive site of ligamentum capitis femoris insertion (red) faces the acetabular ventral margin (+). The middle of the diaphysis is invisible due to chondrocyte maturation/ossification (f1,2). Scale bars, 0.5 mm for 1 s, 1 mm for 2 s, 0.25 mm for 3 s–5 s. (Online version in colour.)

We first examined the embryonic morphology of hindlimb skeletons among extant saurians (electronic supplementary material, figure S2; figure 2). All individuals were aligned with similar orientations with respect to the fibulae (lower limb element), the distal end of the femora, and the acetabula (figure 2*i*). Thus aligned, we compared the orientation of the proximal end of the femora among developmental stages and among species.

In the incipient stage of alligator femoral head morphogenesis (FG 15), a small structure appears at the centre of the proximal end of the femoral anlagen which presumably corresponds to the postero-medial tuber (PMT; figure 2*a*). In the following stages, two additional condensations appear in rostral and caudal positions; these correspond to the antero-medial tuber (AMT) and the ‘greater trochanter’ (GT; cf. not homologous with mammalian GT) (figure 2*b*). Once these formed, the femur's overall shape is similar to the adult condition (figure 2*c*). Turtle femora develop in a similar way with respect to these features (electronic supplementary material, figure S2d-f, electronic supplementary material, SI.2). Thus, this condition can be interpreted as the ancestral embryonic state from Archelosauria to Archosauria nodes, the clade including crocodylians and dinosaurs.

**Figure 3.**
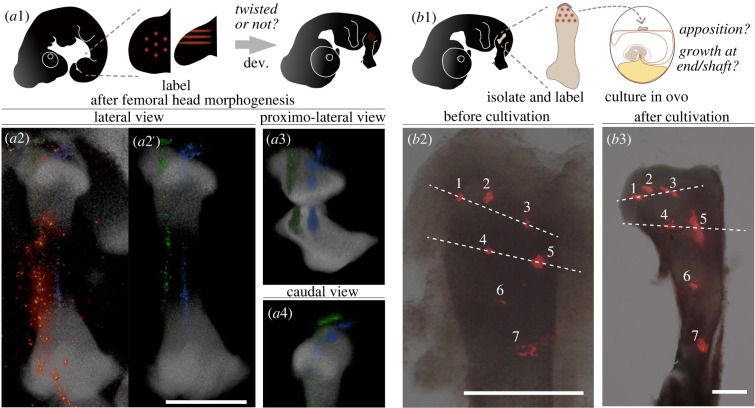
Neontology: morphogenesis of the avian femoral head overhang. (*a*) Labelling experiment to detect/deny ontogenetic torsion of the femoral region. (*a*1) illustrates the scheme; well prior to femoral head morphogenesis (HH stage 21–22, left), hind limb bud mesenchyme was labelled with fluorescent ink linearly in parallel (red lines); then let embryo continue developing to form its femoral head (right). (*a*2–4) shows ink distribution (red-orange, with clearly labelled lines virtually highlighted in green and blue) in the skeleton (grey) at the stage with almost adult orientation of the femoral head in lateral (*a*2), proximo-lateral (*a*3), and caudal (*a*4) views of the representative individual. All are demonstrated in pseudo-colours. (*b*) Labelling experiment to show the detailed morphogenesis of medial overhang formation of the avian femoral head, showing that the avian femoral head develops via deflected growth at the metaphysis, neither via apposition of surrounding cells nor via mediad growth within the epiphysis. (*b*1) illustrates the scheme; The femoral anlagen were isolated from a chicken embryo with hip joint interzone attached (thick line on the proximal end of the isolated femoral anlagen), labelled by fluorescent ink, then cultured on a chorio-allantoic membrane of a host egg (different individual from the femur donors). Ink distribution was shown just after labelling (*b*2) and after culture (*b*3) in extensor-flexor direction view. Arbitrary numbers are assigned for each ink point. Two dotted lines were set between 1&3 and 4&5. Scale bars, 0.5 mm. (Online version in colour.)

**Figure 4.**
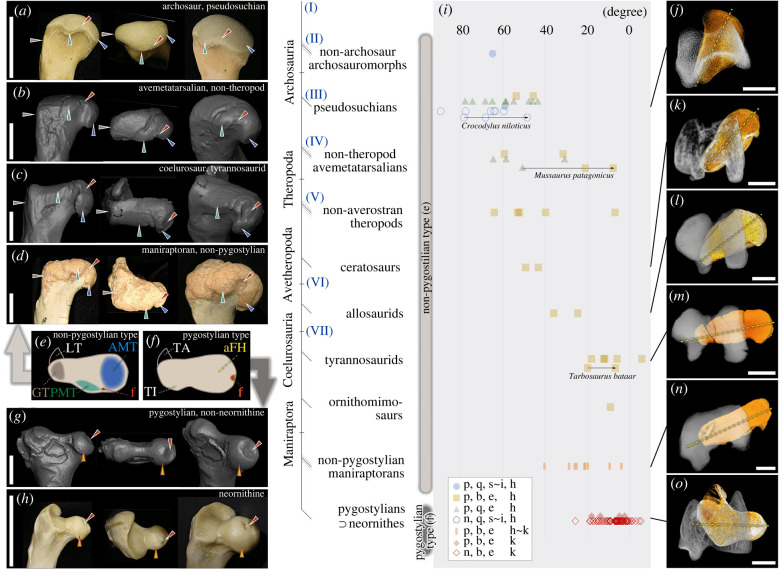
Palaeontology: the morphological transformation of the archosaurian femur. (*a–d*, *g–h*) Left is caudo-medial to caudal view (depending on their post-hatching posture). Middle is proximal view. Right is proximo-medial to proximo-medio-caudal view (ditto). (*a*) *Crocodylus niloticus* (YPM HERR 010809), (*b*) *Mbiresaurus raathi* (NHMZ 2222 [38]), (*c*) *Tyrannosaurus rex* (FMNH PR 2081), (*d*) *Velociraptor mongoliensis* (IGM100.986), (*g*) *Hesperornis regalis* (YPM VP 001476, mirrored), (*h*) *Gallus gallus* (SE). (*e*) and (*f*), schematic generalized morphologies of the proximal end for non-pygostylians and pygostylians, respectively. Corresponding evolutionary grades are indicated by grey bars right to the cladogram for each type. Scale bars, 2 cm for (*a*), (*b*), (*d*), (*g*), (*h*); 20 cm for (*c*). Roman numerals (I–VII) represent evolutionary grade/clade for easier readability of the main text. Arrowheads are: green with dotted frame, PMT; blue, AMT; red, fovea; grey, GT; yellow with red frame, ‘avian’ (pygostylian-type) femoral head. (*i*) shows measured angles between the proximal and distal end of the femora (See Material and Methods for the definition) averaging each genus except for those with ontogenetic series (*C. niloticus, M. patagonicus, T. bataar*). Captions: specimen age, palaeontological/neontological (p/n); locomotory modes; quadru-/bi-pedal (q/b), sprawling∼intermediate∼erect posture (s/i/e), hip-/knee-driven (h/k). In (*j–o*), orthographic distal view of the right (or mirrored left) femora, in which femoral head orientations are comparable to the proximal view of the left femora. The proximal ends are depicted in orange with dotted lines showing their long axis, and the distal ends in grey with their axis (not shown) set strictly horizontally. (*j*) *Crocodylus johnstoni* (QMJ 47916, mirrored left), (*k*) *Masiakasaurus knopfleri* (MAD-05467-2, right), (*l*) *Allosaurus fragilis* (UMNH VP 7884, mirrored left), (*m*) *Teratophoneus curriei* (UMNH VP 16690, right), (*n*) *Falcarius utahensis* (UMNH VP 14666, right), (*o*) *Gallus gallus* (PJB, mirrored left). Scale bars, 1 cm for (*j*), (*k*), (*n*); 5 cm for (*l*), (*m*); 0.5 cm for (*o*). (Online version in colour.)

In the incipient stage of quail femoral head morphogenesis (HH 26), similarly, a small structure appears at the centre of the proximal end of the femoral anlagen (figure 2*d*). At the following stage (HH 28; figure 2*e*), an additional condensation appears on the caudal side of the proximal end. This condensation becomes the trochanter minor (TI). In addition, a second, faint condensation appears on the rostro-lateral side of the proximal end. This condensation subsequently expands to become the trochanter major (TA) (figure 2*e,f*). From HH stage 28 on, the definitive avian femoral head begins to form: between HH 28 and 30, the proximo-medial portion of the proximal end grows directly towards the acetabulum, which is a similar orientation of the adult femoral head (cf. figure 4*o*, chicken); no torsion of the femoral anlagen was apparent. We confirmed the lack of torsion using ink-labelling experiments (figure 3*a*). Well prior to femoral head morphogenesis (HH stage 21–22), we labelled hindlimb mesenchyme with parallel lines of fluorescent ink and let the embryos continue developing (figure 3*a*1). The ink lines remained parallel as the femoral head offset developed (figure 3*a*2–4). In well-labelled specimens, they ascended from the distal half of the femur, penetrated the trochanter major (TA), then turned medially above the femoral head (figure 3*a*4). To examine continued femoral morphogenesis after HH stage 30, we used a different system: femoral anlagen were isolated from chicken embryos, labelled with fluorescent ink, and cultured in a host egg (figure 3*b*1). In such a culture system, isolated chicken femoral anlagen develop almost normal femoral heads with a short/small size [35]. Ink distribution after culture indicates that the differential growth of the fibular (i.e. lateral) side enhances the medial overhang of the femoral head (evident in the change in angle between lines connecting points 1&3 and 4&5 in figure 3*b*2,3). This suggests that such mediad deflection is underlain by a differential elongation of the growth plate. Long bone elongation, in general, is mediated by mechanical stimuli from muscle contraction (e.g. [36]), which thus explains why mechanical stimuli are necessary to elongate the chicken femoral head offset to the normal length in further development [37].

These embryological results suggest that the medial overhang of the avian femoral head develops owing to the medial-ward growth of the proximal end and this morphogenetic process is the main difference-maker to differentiate the apomorphic dinosaurian/avian femoral head from the plesiomorphic state, although subsequent morphogenesis may affect the femoral head/neck shape slightly. These considerations are consistent with the growth hypothesis.

### (b) Palaeontology supports the torsion hypothesis

We examined the archosaurian fossil record to understand the detailed morphological transformations associated with the origin of the greater overhang of the dinosaur-type femoral head.

We began with a comparison of the morphology of the proximal end of the femur (figure 4*a–h*). In crocodylians (figure 4*a*), the ‘greater trochanter’ (GT) is on one side of the proximal end, the antero-medial tuber (AMT) on the opposite side, and the postero-medial tuber (PMT) between them. The osteological correlate for the ligamentum capitis femoris attachment (the fovea) is located between the AMT and the PMT. We traced the homologues of these bony tubercles and scar, maintaining the same relative positions to each other and with the long axis of the proximal end up to non-pygostylian maniraptorans (figure 4*a–d*, schematized in *e*)—that is, long after the apomorphic femoral overhang evolved. This indicates that the gross morphology of the proximal end of the femur was conservative for a phylogenetic period sufficiently wide enough to capture the acquisition of dinosaurian femoral head overhang (see below).

Next, we analysed the evolutionary transition of femoral head long-axis orientation relative to the distal end (electronic supplementary material, table S1). Previous studies (e.g. [13,15,19,25,39–48]) described the evolutionary transition of the femoral head orientation as follows. The femoral head of plesiomorphic saurians (grade ‘I’ in figure 4) had a small angle with the distal end reference (the intercondylar line). Among early archosauriforms (grade ‘II’), the angle increased (i.e. anteverted); this state is present in archosaurs as well among crocodylian-line one (pseudosuchians; up to extant crocodylians; clade ‘III’) and among early bird-line one (avemetarsalians; grade ‘IV’). Near the origin of Tetanurae (close to node ‘VI’), the angle decreased (i.e. retroverted; to be nearly parallel with the intercondylar line and more medially oriented in reconstructed post-hatching posture); this state is retained in living birds. Our results were consistent with these studies. In non-theropod archosauriforms (grade ‘II’–‘V’; e.g. *Euparkeria*, pseudosuchians including extant crocodylians, *Marasuchus*, silesaurids and sauropodomorphs), the orientation of the proximal end long axis ranged 30–90° (figure 4*i,j*) against the ‘distal end axis’ (a more reliable distal end reference; see Material and Methods). This angle gradually decreased to 5–65° in non-coelurosaurian theropods (around node ‘VI’; figure 4*i,k,l*), and to −5–40° in coelurosaurs including neornithines (clade ‘VII’; figure 4*i,m–o*). Such an evolutionary transition of the femoral head angle is consistent with the previous assumption that the femoral head of early avetheropods (near ‘VI’) articulated with a more rostro-medial orientation during locomotion, versus fully medial in early coelurosaurians (near ‘VII’) (e.g. [16]). Additionally, this assumption is biomechanically consistent with the transformation of the supra-acetabular crest's extent in lateral view, from the rostro-dorsal acetabular margin in early dinosaurs to more dorsal acetabular margin in early coelurosaurs [49,50].

In summary, during the transition from early archosaurs to early maniraptorans, the angle of anteversion gradually decreased (figure 4*i–o*) with the gross morphology of the proximal end conserved (figure 4*a–e*) in adults, which derived the mediad overhang. During this transition, evolutionary torsion is interpreted to have occurred proximal to the diaphyseal insertions of *Mm. caudofemorales (CF*) and *Mm. pubo-ischio-femoralis internus* (*PIFI*), because the positional relationship is nearly constant among the distal end of the femur and these insertions [13,24]. The mediad overhang would be enhanced by femoral neck elongation (electronic supplementary material, SI.3.2).

**Figure 5.**
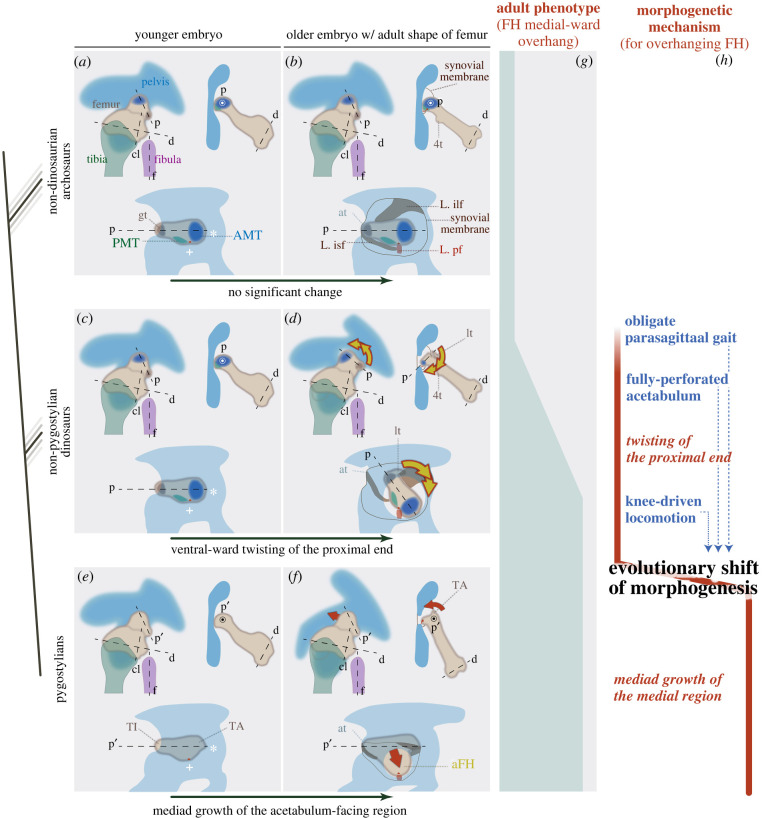
Summary: the evolutionary shift in morphogenetic process of the dinosaurian femoral head overhang while effectively maintaining the resultant phenotype. Top (*a,b*) is the plesiomorphic developmental process inferred for non-dinosaurian archosaurs (e.g. extant crocodylians); Middle (*c,d*) is that for non-pygostylian dinosaurs; Bottom (*e,f*) is that for pygostylians (including extant birds). Left ontogenetic stages (*a,c,e*) represent the younger ones, in which all tuberosities have arisen but just before the incipient morphogenesis of medial overhang (in dinosaurs). Right ontogenetic stages (*b,d,f*) represent the older ones, in which almost adult shapes of the femora have developed (i.e. after morphogenesis of the medial overhang in dinosaurs). Dotted line ‘p’, the axis of the proximal end; dotted line ‘d’, that of the distal end; dotted line ‘cl’, that of condylus lateralis; dotted line ‘f’, that of fibular shaft. It is still controversial whether the axes of the proximal end correspond between non-pygostylian dinosaurs (dotted line p) and pygostylians (dotted line p’). (g) is a graphical representation of the mediad overhanging degree of the proximal ends of the femora (regardless of their homology and morphogenetic mechanism) in adults. (*h*) denotes the morphogenetic process for the medial overhang of the proximal end of the femora (twisting in non-pygostylian dinosaurs, yellow arrows in d; mediad growth of the medial region in pygostylians, red arrow in *f*), highlighting its evolutionary shift while almost maintaining the phenotype of femoral head mediad overhanging. The probable prerequisites for such morphogenetic evolution are listed in blue letters. aFH, ‘avian’ (pygostylian-type) femoral head; AMT, antero-medial tuber; at, antitrochanter; GT, great trochanter; lt, lesser trochanter; L.ilf, ligamentum ilio-femoris; L.isf, ligamentum ischio-femoris; L.pf, ligamentum pubo-femoris (= ligamentum capitis femoris); PMT, postero-medial tuber; TA, trochanter major; TI, trochanter minor; 4t, fourth trochanter;+ and *, ventral and rostral margins of the acetabulum, respectively. (Online version in colour.)

On the basis of these results, we reconstructed femoral head morphogenesis of non-pygostylian theropods. In this evolutionary grade, based on the position of ligament capitis femoris, the relative positions among parts of the incipient embryonic hindlimb skeleton would have remained similar to that of alligators (figure 5*a,c*; see electronic supplementary material, SI.3.1 for rationales). However, in adults, the AMT must be oriented more or less towards the acetabulum (i.e. medially) for functional articulation. This orientation has been convincingly reconstructed (e.g. in coelurosaurs) on the basis of trabecular bone orientation inside femoral head, which is remodelled in reaction to mechanical stimuli in post-hatching locomotion [51]. Therefore, the proximal end of the femur must have re-oriented during ontogeny, from more rostrally directed to more medially directed (figure 5*c,d*). However, such a femoral head re-orientation could not be accomplished by an internal rotation of the whole femur alone, because, if so, the resultant deviation of the lower leg from parasagittal planes would abnormally disrupt bipedal locomotion. Instead, femoral head re-orientation must have been brought about by femoral torsion during ontogeny (figure 5*c,d*). Empirically, femoral torsions can be partially observed in post-hatching stages [19] (figure 4*i*). Given that the positional relationship was conservative in the incipient embryonic hindlimb skeleton (electronic supplementary material, SI.3.1), the extent of ontogenetic twisting reached a maximum in early coelurosaurs (peramorphism; figure 4*i*).

We infer that this torsion was brought about by mechanical stimuli of muscle contraction during ontogeny. The analogous retroversion of the mammalian femoral head (e.g. [52–57]) and the twist of avian metatarsal I [58] are regulated by mechanical stimuli from muscular contraction during late-stage morphogenesis. We suggest that the orientation of the archosaur femoral head is similarly susceptible to mechanical stimuli. We found that one of four femora of a conjugated twin embryo of a crocodylian was abnormally abducted (certainly caused by spatial constraints on two bodies within one egg) and its femoral head orientation was exceptionally anteverted (electronic supplementary material, figure S8). Since all four femora must have shared the effectively same backgrounds in physiology and genotype, it is reasonable to attribute this angular difference to mechanical factors.

The fossil record demonstrates muscle attachment evolution, indicating changes in mechanical stimuli which would bring about twisting morphogenesis (i.e. retroversion) of non-pygostylian theropods' femoral head. The region where evolutionary torsion occurred (i.e. immediately proximal to *Mm. CF* and *Mm. PIFI* insertions; see above) is inserted by *M. iliotrochantericus caudalis* (*ITC*). From early archosaurs to early coelurosaurs, this muscle increased torque on the femoral neck region towards protracting and medially/internally rotating direction (electronic supplementary material, SI.3.3). Such a contrast is evident in the embryonic muscle shape between non-dinosaur archosaurs (alligator) and coelurosaurs (quail) [59], thus the biomechanical condition of adults in this regard would have been established in embryonic stages. Consequently, the actions of this muscle would apomorphically promote femoral twisting (i.e. retroversion) during ontogeny of non-pygostylian theropods, as do the similarly acting muscles in human ontogeny [56]. This hypothesis explains homoplastic covariation from a morphogenetic viewpoint: the combination of femoral head retroversion and *M.ITC*-related skeletal changes appeared several times independently in major archosaurian clades [14,19,25,60]. We infer that the evolution of the *M.ITC's* configuration, consequent femoral twisting in ontogeny, and other musculo-skeletal changes accompanying them was functionally advantageous for (thus would have been ‘least-resisted’ by) their locomotory biomechanics (hip-driven erect posture) especially to theropods' plesiomorphic one (hip-driven bipedalism) (electronic supplementary material, SI.3.4).

These considerations suggest that the medial overhang of the femoral head in more crownward theropods developed owing to an ontogenetic twisting of the proximal end of the femur (i.e. retroversion in ontogeny) which plesiomorphically projected more rostrad, and this morphogenetic process is the main difference-maker to differentiate the apomorphic dinosaurian/avian femoral head from the plesiomorphic state. This is consistent with the torsion hypothesis, but apparently contradicts the growth hypothesis. Therefore, there is a conflict between neontology- and palaeontology-derived hypotheses.

### (c) Reconstruction of morphogenetic evolution: an integrative solution

To reconcile these apparently disparate hypotheses, we propose a scenario in which the mode of morphogenesis shifted from twisting to mediad growth while the adult phenotype remained effectively constant (figure 5*g,h*) during avialan evolution, as originally implied by ref. [23] in early maniraptorans—however, aspects of our scenario differ (electronic supplementary material, SI. 7.2). Canonically, reconstructions of morphogenetic evolution have been hypothesized by extrapolating that (apparently) homologous states develop in the same manner among extinct and extant taxa (for this study, mediad overhang of the femoral head). However, morphogenesis can change while adult phenotypes remain static [61–68].

Potentially, the same developmental processes among different species can be misinterpreted as disparate, especially when they occur in different situations (e.g. different timings; heterochrony). Hence, in the following sections, we test this scenario in more detail, highlighting the difference in the morphogenetic mechanisms of these two processes (retroverting torsion of non-pygostylians and mediad growth of pygostylians).

### (d) Additional evidence and narrowing of the phylogenetic window of morphogenetic shift

In embryos, the presumptive insertion sites of the ligamentum capitis femoris (face the ventral margin of the acetabulum during femoral head morphogenesis in gecko, alligator and quail (electronic supplementary material, figure S2, figure 2, figure 5*a,b,e,f*). Therefore, this embryonic state can be inferred as continually present from the saurian common ancestor to crown-group birds. In extinct non-pygostylian archosaurs, the osteological correlate for this ligament (the fovea) is located between the AMT and the PMT off the tip of the femoral head long axis [15], whereas it is at the centre of the femoral head apex since early pygostylians (*Confuciusornis* [69]; enantiornithines [70–73]; and early euornithines [74,80,81] ) (figure 4*a–h*). Thus, during the evolution to pygostylians, the fovea seemingly relocated to the centre of the femoral head [82] as though the ligament itself shifted its insertion (figure 4*e* versus *f*; electronic supplementary material, SI.4). However, instead of ligamentous repositioning, it is more consistent to attribute this change to the evolutionary shift of femoral head morphogenesis from torsion to mediad growth: the proximal end including the fovea changed its orientation during ontogeny in the former but not in the latter (figure 5*c,d* versus *e,f*).

The articulation of the pelvic antitrochanter is also consistent with this scenario. The antitrochanter typically articulates with GT homologues (electronic supplementary material, SI.7.1.1) in crocodylians and neornithines (figure 2*c*5,*f*5) whereas it does not in non-pygostylian theropods [15,51,83] (compare figure 5*b,d,f*). This contrast is best explained by ontogenetic twisting only in non-pygostylian theropods, resulting in a greater distance between GT and antitrochanter.

### (e) Possible triggers for evolutionary change in morphogenesis—muscular contraction

We infer that the femoral twisting in non-pygostylian theropods’ ontogeny was brought about by mechanical stimuli from the *M.ITC's* contraction (see above). For the *M. ITC* to effectively exert mechanical stimuli, other muscles would need to stabilize the distal portion of the femur. Otherwise, not only the proximal end but also the embryonic femur as a whole would rotate medially. To prevent this, *Mm. iliotibiales (IT)* and *Mm. CF* would work most antagonistically as stabilizers (i.e. exert a laterally rotational moment) (see [17] for adult moment arms).

Early in maniraptoran evolution, gaits transformed from more hip-driven to more knee-driven with numerous anatomical changes in the hip musculoskeletal system and this evolutionary trend continued up to the base of Pygostylia (e.g. craniad expansion of pre-acetabular ala of the ilium; electronic supplementary material, SI.6). However, evolution to such a more ‘knee-driving’ locomotory system would gradually attenuate the stabilizing effect (e.g. of the cranial part of *Mm.IT*) against ‘twisting muscle’ (*M.ITC*) [17], becoming lesser compatible with the ontogenetic twisting in morphogenesis (electronic supplementary material, SI.6). Therefore, the evolutionary shift in morphogenetic mechanism (from ‘stabilizing musculature’-dependent to -independent) would be a ‘solution’ allowing a shift to more knee-driven locomotion while keeping mediad overhang of the femoral head and the buttress erect posture necessary for theropod-style locomotion.

### (f) Possible triggers for evolutionary change in morphogenesis—acetabular size

For the ontogenetic twisting to occur during morphogenesis, the acetabulum of embryo to juvenile stages must be wide enough for the AMT to rotate within it. As expected, we find the archosaurian acetabulum to be much wider relative to the proximal end of the femur in the plesiomorphic state than in the derived state in alligator and quail embryos, respectively (electronic supplementary material, figure S3). Additionally, in adults of extinct dinosaurs the relative size of acetabular perforation gradually diminished in size towards ornithurans [11]. Embryonic joint spaces have special mesenchymal layers called the interzone (IZ), which spans the acetabulum in embryonic saurian hip joints, and IZ-expressed molecules (canonical Wnt ligands) cause the perforation of the avian acetabulum [84]. Therefore, that the evolutionary reduction of adults' acetabular size probably reflects a reduction of the IZ range and thus, that of the acetabulum of embryos. This evolutionary reduction of relative size in the embryonic acetabulum could require the evolutionary change of morphogenetic mode, and/or the latter could allow the former.

The attenuation/loss of femoral ontogenetic twisting had to be compensated by another morphogenetic process to produce a medial overhang of the femoral head to maintain the buttress-erect hip joint. Size reduction of the embryonic acetabulum could have produced the derived morphogenetic mechanism; mediad growth. As mentioned above, IZ distribution in the embryonic hip joint would have decreased in evolution and consequently would result in a change of IZ coverage of the proximal end of the femur; from almost the entire epiphysis to the medial region exclusively (electronic supplementary material, figure S7a,b,e,f). IZ layers in general secrete several molecules that regulate long bone elongation at the growth plate (e.g. [85–87]). Such a change in IZ coverage would have resulted in a deflected expansion of the growth plate towards IZ (electronic supplementary material, figure S7 b versus f), which can be seen in extant pygostylians (figure 3*b*), thereby bringing about pygostylian-type femoral head foramtion. If this scenario is true, gradual size reduction of the embryonic acetabulum was a prerequisite for the evolutionary shift of femoral head morphogenesis. We infer that the acetabular size reduction was more feasible (i.e. ‘less-resisted’) in maniraptorans because of their knee-driven locomotory biomechanics over their hip-driven ancestors (electronic supplementary material, SI.6.2).

## 4. Concluding remarks

By analysing embryos of extant relatives and the fossil record, we have reconstructed the complex evolutionary history of the dinosaurian femoral head—a history in which evolutionarily continuous (i.e. apparently homologous) adult morphology was reconstructed to be underlain by differing morphogenetic processes. This highlights the general importance of reconstructing morphogenetic evolution in extinct grades invoking not only neontological embryos but also the fossil record.

Specifically, we hypothesize that coelurosaurian morphogenesis shifted from a peramorphic twisting to mediad growth, while adult phenotype (medial-ward overhang of the femoral head) and general function (enabling buttress erect) remained effectively constant (figure 5). In other words, we suggest that whereas non-pygostylian coelurosaurians developed the femoral head overhang recapitulatively, pygostylians do not (i.e. caenogenetically; *sensu* [61]). Our evidence and considerations suggest that the avian lineage first acquired the derived phenotype along an evolutionary line of least resistance for the ancestral developmental/locomotory system (peramorphic twisting), and secondarily in a more demanding but direct way (mediad growth). Specifically, we posit that the derived morphogenesis likely needs more developmental and locomotory apomorphies as prerequisites (obligate parasagittal gait [6,9]; fully perforated acetabulum, electronic supplementary material, SI.5; acetabular size reduction and more knee-based locomotor system, electronic supplementary material, SI.6). This indicates the importance of considering integrity among morphogenetic and functional circumstances (electronic supplementary material, figure S9)—just as Goethe [88] and Piaget [89] embraced ceaseless reciprocal influences and resultant congruency among organismal parts.

## Data Availability

Access to digital data of the fossil specimens originally collected for this study can be obtained on reasonable requests through contacting the collections management staff of the respective museums where the original specimens are kept. The data are provided in electronic supplementary material [90].
